# Optimized PID Controller Based on Beetle Antennae Search Algorithm for Electro-Hydraulic Position Servo Control System

**DOI:** 10.3390/s19122727

**Published:** 2019-06-18

**Authors:** Yuqi Fan, Junpeng Shao, Guitao Sun

**Affiliations:** School of Mechanical and Power Engineering, Harbin University of Science and Technology, Harbin 150080, China; yuqifan77@163.com (Y.F.); sunguitao86@163.com (G.S.)

**Keywords:** PID controller, electro-hydraulic servo system, beetle antennae search algorithm

## Abstract

To improve the controllability of an electro-hydraulic position servo control system while simultaneously enhancing the anti-jamming ability of a PID controller, a compound PID controller that combines the beetle antennae search algorithm with PID strategy was proposed, and used to drive the position servo control system of the electro-hydraulic servo system. A BAS-PID controller was designed, and the beetle antennae search algorithm was used to tune PID parameters so that the disturbance signal of the system was effectively restrained. Initially, the basic mathematical model of the electro-hydraulic position servo control system was established through theoretical analysis. The transfer function model was obtained by identifying system parameters. Then, the PID parameter-tuning problem was converted into a class of three-dimensional parameter optimization problem, and gains of PID controllers were adjusted using the beetle antennae search algorithm. Finally, by comparing the effectiveness of different algorithms, simulation and experimental results revealed that the BAS-PID controller can greatly enhance the performance of the electro-hydraulic position servo control system and inhibit external disturbances when different interference signals are used to test the system’s robustness.

## 1. Introduction

Electro-hydraulic servo systems (EHSSs) have been widely used in the industry due to their advantages of high response, fast response, good stiffness, and high force [1]. With the development of digital technology, control theory, pattern recognition technology, and electronic technology, EHSSs have become one of the most popular topics in the fields of both scientific research and industrial engineering [2,3]. EHSSs including a position servo control system and a force servo control system are a typical closed-loop torque control system [4,5]. 

Position servo control systems play a major role in marine operating systems, industrial automation systems, and crane systems [6]. Due to the complexity of such control systems, it is considered urgent to develop control technology. Various control methods have been presented, such as the feedback control method [7], tracking control method [8], and adaptive control method [9]. Among these, the PID control system is the most popular control method. Due to its easy control structure, high robustness, and high accuracy, the conventional PID control system is the most widely used in industrial processes [10,11,12].

The chief function of PID controllers is to regulate feedback signals to be as similar to input signals as possible. The control performance of PID controllers is mainly determined by a proportional parameter, an integral parameter, and a differential parameter [13,14]. It has been shown that rapid response and high accuracy of velocity can be obtained by selecting appropriate PID parameters in the control system. Therefore, the method of tuning PID parameters has become a popular research topic, and some significant achievements have been made in recent years [15,16].

Over the past few decades, several methods which can enhance the capacity of obtaining reasonable PID parameters have been announced. One of the most popular methods is the Z-N tuning method [17], proposed by Ziegler and Nichols in 1942. This method is widely used in industry to solve the traditional PID parameter tuning problem [18]. However, the mechanism of the controlled object is complex in actual industrial circumstances; many factors need to be considered, such as high-order duplication, time variation, and non-linearity. Consequently, it is difficult to achieve a perfect control result by using the Z-N tuning method. However, since the method’s publication, it has been extensively developed for tuning PID parameters. 

With the development of artificial intelligence science, artificial intelligence algorithms have been widely used in PID controllers to select reasonable PID parameters, for example the Genetic Algorithm (GA) [19,20], Particle Swarm Optimization Algorithm (PSO) [21,22], Firefly Algorithm (FA) [23,24], Cuckoo Search Optimization Algorithm (CS) [25], Grey Wolf Optimization Algorithm (GWO) [26], Ant Colony Algorithm (ACA) [27], and Bacterial Foraging Optimization Algorithm (BFO) [28]. The Beetle Antennae Search Algorithm (BAS) is an efficient and intelligent search algorithm proposed by Xiangyuan Jiang and Shuai Li in 2017 [29]. It is an evolutionary computation derived from the flocking and swarming behavior, foraging behavior and courtship behavior of beetles. The algorithm not only has the ability of individual recognition and environmental recognition, but also does not need to know the gradient information of the function. Furthermore, its program code is simple to implement, and it has an especially strong ability to obtain the best solution within a stable convergence property. BAS is used in neural network optimization [30], multi-objective energy management [31], investment portfolio problems [32], the prediction of the content of dissolved gases in power transformer oil [33], the determination of Young’s modulus in jet grouted coalcretes [34], prediction of permeability and unconfined compressive strength of pervious concrete [35], multi-objective optimization based BAS-WPT [36], and unmanned aerial vehicle (UAV) sensing and avoidance [37].

To improve the PID controllability of an electro-hydraulic position servo control system, this paper proposes a BAS-PID controller whose parameters are tuned by BAS. To verify the performance of the BAS-PID controller in the electro-hydraulic position servo control system, the controllability of the BAS-PID controller was compared to the controllability of PID controllers using other algorithms under different interference signals. Experimental results showed the superiority of the BAS-PID controller.

## 2. Related Works

BAS is extremely efficient in solving different optimization problems, and the original BAS, as well as BAS variants, have been successfully applied in various scientific and engineering fields. 

Yuantian Sun et al. [30] proposed combination of the back-propagation neural network (BPNN) and BAS, termed the BPNN-BAS model. The BPNN-BAS model was shown to be more effective than multiple regression and logistic regression. Additionally, in another study [34], support vector machine (SVM) was combined with BAS to produce the SVM-BAS model for the determination of Young’s modulus in jet grouted coalcretes. Zongyao Zhu et al. [31] applied BAS in microgrids to reduce environmental pollution and minimize pollutant treatment cost. Tingting Chen et al. [32] proposed the Beetle Swarm Optimization Algorithm (BSO), which combines BAS and PSO. Shengwei Fei et al. [33] developed the BAS based mixed kernel relevance vector regression (BAS-MkRVR) model to calculate the gas content of power systems. Junbo Sun et al. [35] introduced an ESVR-BAS model to predict the PC and UCS in pervious concrete. Xiangyuan Jiang and Shuai Li [36], who are designers of BAS, advanced the research on BAS by releasing tuning parameters to deal with multi-objective optimization. Qing Wu et al. [37] invented a new path algorithm called the Obstacle Avoidance Beetle Antennae Search Algorithm (OABAS), which is used for global path planning for UAVs. Meijin Lin et al. [38] proposed a hybrid PSO-BAS algorithm, while the same lead author together with Qinghao Li [39] used PSO-BAS to distribute economic loads in power systems. Qinhao Li [40] used BAS to solve optimal scheduling problems of a wind–PV–hydro–storage power complementary system. Chen Wang et al. [41] employed BAS to enhance the precision of the evaluation of straightness error. Table 1 provides a brief comparison among BAS variants.

In [42] Yinyan Zhang, Shuai Li, and Bin Xu summarized and analyzed the convergence of BAS and its applications, which provided further theoretical proof of its extreme convergence ability.

For a complex optimization problem with multiple parameters, the complexities of the solution space increase with increasing dimensionality, which may cause low convergence speed. For potential extensions and future work, in order to obtain a more accurate solution, the structure of BAS should be improved. Further research may consider an encoding scheme to introduce better diversity into the solution search space of BAS to raise the convergence of the algorithm. In this study, we will apply advanced BAS to solve more practical questions about electro-hydraulic systems. Table 2 shows the uniqueness of BAS among algorithms including PSO [32,37,38,39], GA [39], FA, BA [42], and the Artificial Bee Colony Algorithm (ABC) [37]. 

From Table 2, it can be determined that BAS not only has abilities of individual recognition and environmental recognition but also allows the balance in exploratory stages, which shows that it is necessary to use BAS to develop better sensing systems.

## 3. Electro-Hydraulic Position Servo Control System

### 3.1. Working Principles of the System

**Figure 1:**
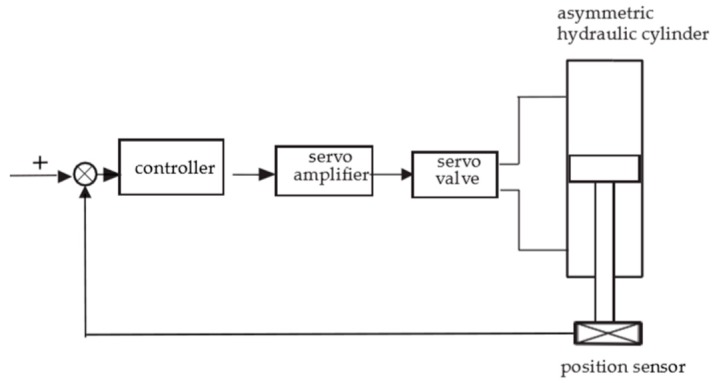
Block diagram of the electro-hydraulic position servo control system.

The electro-hydraulic position servo system studied in this paper is chiefly composed of a hydraulic cylinder, an electro-hydraulic servo valve, a servo amplifier, a position sensor, and some other parts. A schematic diagram of the system is displayed in Figure 1. 

The working principle of the position servo system is characterized as follows. Firstly, the voltage signal is transformed into an electric current signal by the servo amplifier and then input to the servo valve, and the slide valve of the main valve will be moved depending on the signal of the servo valve. Then, the flow rate of the hydraulic cylinder is influenced by the movement of the slide valve. The piston rod is linked to the position sensor. Finally, depending on analogue–digital conversion, the signal will be fed back to the controller of the control system. Overall, the error between the actual signal and the target signal will be constantly decreased by the position servo control system.

In practical engineering problems, due to complexities of the structure, manufacturing errors, large delay time, inaccurate measurement, and other factors, it is difficult to accurately describe the whole working process by building a precise mathematical model. There is not a determined mathematical model, and therefore the chief aim of this paper is to make the electro-hydraulic servo system model as similar to the really original model as possible. 

### 3.2. Model of the Electro-Hydraulic Position Servo Control System

The electro-hydraulic position servo system studied in this paper is an electro-hydraulic flow servo valve which controls cylinder structure. The electro-hydraulic servo valve is used as an electro-hydraulic transfer apparatus, and is put into a lower input signal to obtain effective hydraulic pressure energy and to achieve the aim of control for hydraulic systems.

(1) $$K_{v}=\frac{x_{v}(s)}{I(s)}$$

where *I* is the output current, *x_v_* is the spool displacement of the main valve, and *K_v_* is the flow gain of the electro-hydraulic servo valve. 

The input voltage signal in the system is transformed into an electric current signal by the servo amplifier. Then the electric current signal is passed into the pilot valve. Therefore, the output current of the servo amplifier can be seen as proportional to the input voltage, which can be regarded as a purely proportional stage and its mathematical model can be expressed as

(2) $$K_{a}=\frac{I(s)}{U(s)}$$

where *I* is the output current, *U* is the input voltage, and *K_a_* is the amplification coefficient of the amplifier.

A position sensor which has the advantages of small size, low weight and fast response is used in the feedback stage. The sensor acquires the position signal by calculating and converting it into a feedback signal. The response frequency of the position sensor is much higher than the response frequency of the whole system. Hence, the process can be seen as a purely proportional stage, and the transfer function can be expressed as

(3) $$U_{f}=K_{m}x_{P}$$

where *U_f_* is the output voltage, *x_P_* is the piston displacement, and *K_m_* is the magnification coefficient of the position sensor.

The linearized load flow equation, hydraulic cylinder flow continuity equation, and the force-balance equation of the hydraulic cylinder need to be calculated to illustrate the model of the asymmetrical hydraulic cylinder. The linearized load flow equation from the servo valve to the hydraulic actuator can be written as

(4) $$q_{L}=K_{q}x_{v}-K_{c}p_{L}$$

where *q_L_* is the load flow of the servo valve (m^3^/s), *K_q_* is the flow gain coefficient (m^2^/s), *x_v_* is the open amount of valve spool (m), *K_c_* is the flow pressure coefficient (m^5^ /(N·s)), and *p_L_* is the loading pressure of the cylinder (Pa).

The flow continuity equation of the hydraulic cylinder can be expressed as

(5) $$q_{L}=A\frac{dy}{dt}+C_{tc}p_{L}+\frac{V_{t}}{4\beta e}\frac{dp_{L}}{dt}$$

where *A* is the effective area of cylinder piston (m^2^), *C_tp_* is the total leakage coefficient (m^5^ /(N·s)), *V_t_* is the total volume of the cavity (m^3^), *β_e_* is the effective bulk modulus (Pa).

The force balance equation of the hydraulic cylinder without considering the friction and quality of the oil can be expressed as

(6) $$Ap_{L}=m\frac{d^{2}y}{dt^{2}}+\beta_{c}\frac{dy}{dt}+Ky$$

where *m* is the colligation quality of the cylinder piston discreteness (kg), *β_c_* is the viscous damping coefficient of the piston and load (N/(m/s)), *K* is the spring stiffness (N/mm), and *y* is the displacement of the piston (m).

Equations (4) to (6) represent dynamic models of the hydraulic cylinder. Their Laplace transforms can be written as

(7) $$Q_{L}=K_{q}X_{v}-K_{c}P_{L}$$

(8) $$Q_{L}=A_{1}Ys+C_{tc}P_{L}+\frac{V_{t}}{4\beta e}P_{L}s$$

(9) $$AP_{L}=ms^{2}Y+B_{c}sY+KY$$

The transfer function of the position servo control system can be deduced by Equations (1) to (9)

(10) $$G(s)=\frac{\frac{K_{q}}{A_{}}X_{v}-\frac{\left(K_{c}+C_{tp}\right)}{Av}(1+\frac{V_{t}}{4\beta_{e}(K_{c}+C_{tp})}s)P_{L}}{\frac{mV_{t}}{4\beta_{e}A_{}^{2}}s^{3}+(\frac{m(K_{c}+C_{tp})}{A_{}^{2}}+\frac{V_{t}}{4\beta_{e}A_{}^{2}})s^{2}+(1+\frac{B_{c}(K_{c}+C_{tp})}{A_{}^{2}}+\frac{KV_{t}}{4\beta_{e}A_{}^{2}})s+\frac{K(K_{c}+C_{tp})}{A_{}^{2}}}$$

## 4. BAS-PID Control System

### 4.1. Beetle Antennae Search Algorithm

BAS is a meta-heuristic intelligent optimization algorithm based on group optimization. The position of each beetle represents an achievable optimized solution. Beetles use antennae on two sides of their body to find food resources. When the antenna on one side is closer to food, the food odor received by the antenna is stronger, and the individual will move to that side. This new meta-heuristic algorithm BAS taking inspiration from detecting and searching behavior of longhorn beetles.

We denote the strength of the food odor at position *x* to be the value at point of the optimized function.

To explore the initial unknown environment, the initial beetle searching is supposed to move randomly in any direction. A vector with a random direction can be built to be representative and normalize in spaces of any dimension. A random searching direction can be normalized as

(11) $$\vec{b}=\frac{rnd(dim,1)}{\left\|rnd(dim,1)\right\|}$$

where *rnd(.)* denotes a random function and dim represent the dimensions of the position.

Beetles do not know the precise location of food when foraging. They use both antennae to detect the food odor and move in the direction of the odor. The positions of the right and left antenna can be obtained as

(12) $$x_{r}=x^{t}+d^{t}\cdot \vec{b}$$

(13) $$x_{l}=x^{t}-d^{t}\cdot \vec{b}$$

where *t* is the iteration number, *x_r_* denotes the position of the right antenna, *x_l_* denotes the position of the left-antenna, *x^t^* is the position of the beetle, and *d^t^* is the sensing length of the antennae.

The beetle will move to the left if the left antenna receives a stronger scent than the right antenna; otherwise, it will move to the right. 

The beetle chooses its search behavior based on the direction of the detected odor. Thus, we can determine the next position of a beetle by judging the strength of the odor.

The next position of the beetle can be determined by

(14) $$x^{t}=x^{t-1}+c\cdot \delta^{t}\cdot \vec{b}\cdot sign(f(x_{r})-f(x_{l}))$$

where *δ^t^* is the step size of searching, *sign(.)* represents a sign function, *f(.)* is an optimized function, and *c* is the direction of beetle movement. If the aim is to find the maximum value, *c* = −1. If the problem is to find the minimum value, *c* = +1. 

The sensing length of the antennae *d^t^* and the step size of searching *δ^t^* can be updated as

(15) $$d^{t}=0.95d^{t-1}+0.01$$

(16) $$\delta^{t}=0.95\delta^{t-1}$$

The iterative process of the beetle antennae search algorithm can be presented as follows:

Step 1: Set the maximum number of iterations *t_max_*. Randomly initialize N beetle positions *X_i_*(*I* = 1, 2, …, N). Define all beetle antennae search algorithm parameters, including the initial step size of searching *δ* and the initial sensing length of the antennae *d*. Define the optimized function. Set direction of the beetle movement *c* according to the optimization purpose of the optimized function.

Step 2: Generate random searching directions using Equation (11). 

Step 3: Update the positions of the right antennae of beetles using Equation (12). Update positions of the left antennae of beetles using Equation (13).

Step 4: Modify beetle positions according to Equation (14). 

Step 5: Calculate all feasible solutions and compare their fitness values to determine the optimal solution in the current generation. After comparing the current minimum fitness value with the previous minimum fitness value, update the global optimum solution if there is a better solution.

Step 6: Update the sensing length of antennae using Equation (15). Update the step size of beetles using Equation (16).

Step 7: Update the number of iterations *t* = *t* + 1 and return to Step 2. Repeat until *t* = *t_max_*.

Step 8: Output the global optimum solution. 

In order to explain the BAS more clearly, we have detailed the steps of the BAS in Algorithm 1. 


**Algorithm 1: BAS**
**Input:** Set the maximum number of iterations *t_max_*. Define the evaluation function *f(.).* Randomly set *N* beetle positions *x^t^_i_*(*i* = 1, 2, …, *N*). Set *t* = 0. Set the value of c according to the optimization purpose. Record the initial sensing length of antennae *d^0^*, the initial step size *δ^0^*_._ Record the initial optimum solution, *x_best_*, and the initial optimum value, *g_best_*_._**Output:***x_best_*, *g_best_*.1: **while** (*t* < *T*_max_)2:  **For**
*i* = 1: N3:  Update the searching direction *b_i_* using Equation (11) 4:  Update the right antenna position *x_ri_* using Equation (12) 5:  Update the left antenna position *x_li_* using Equation (13) 6:  Update the next position *x_i_^t+1^*of the beetle *x_i_^t^* using Equation (14).7:  **End for**8:  **For**
*i* = 1: N9:   Calculate the function value *f*(*x_i_^t+^*^1^) of *i*th beetle.10:   **If**
*f*(*x_i_^t+^*^1^)is better than *g_best_*
11:      *x_best_* = *x_i_^t+^*^1^12:      *g_best_* = *f*(*x_i_^t+^*^1^)13:   **End if**14:     **End for**15:  Update the sensing length of the antennae *d^t^* using Equation (15).16:  Update the step size of searching *δ^t^* using Equation (16).17:  *t* = *t* + 118: **End while**

### 4.2. PID Control System

PID controllers which have high efficiency and robust performances, are linear controllers. Thanks to their easy control structure, high robustness, and high accuracy, PID controllers are commonly used in engineering to enhance both transient and steady-state behaviors. It has been shown that rapid response speed and high controllability can be obtained by determining appropriate PID parameters. PID controllers are widely used in different control systems. There are three parameters in a PID controller, namely the proportional parameter *K_p_*, the differential parameter *K_d_*, and the integral parameter *K_i_*. These three parameters have dramatic effects on the performance of control systems: *K_p_* affects the response speed of the system, *K_d_* affects dynamic performance, and *K_i_* affects the steady state error. The current PID controller can be divided into two main modes: the continuous form and the discrete form.

The continuous form of the PID controller is described as

(17) $$u(t)=K_{p}e(t)+K_{i}\int_{0}^{t}e(t)dt+K_{d}\frac{de(t)}{dt}$$

where *K_p_* is the proportional parameter, *K_d_* is the derivative parameter, *K_i_* is the integral parameter, *u*(*t*) is the output of the PID control signal, and *e(t)* is the system error signal.

Let the sampling instant replace continuous time. Let the integral item and differentiation item be discretized, and the discrete form of PID controller is written as

(18) $$u(k)=K_{p}e(k)+K_{i}T\sum_{k=0}^{m}e(k)+\frac{K_{d}}{T}[e(k)-e(k-1)]$$

where *T* is the sampling period, *k* is the sampling number, and *m* is the total number of sampling times.

### 4.3. System Evaluation Function

The value of the evaluation function is used to measure the speed of the response of the control system. It is necessary to select an evaluation function before the optimization.

The evaluation function of the control system includes the integration of the absolute value of error (IAE), the integral of time multiplied by the absolute value of error (ITAE), the integral of the square value of error (ISE), the mean of the square of the error (MSE). For the PID control system, the objective function based on a single measurement value only evaluates a part of the system. 

The IAE, which give importance to the absolute error, only takes single factors into account. The IAE is often used in the digital simulation of systems, since it is somewhat difficult to obtain the absolute value of the error in analytic form. So, in order to comprehensively evaluate the performance of electro-hydraulic servo system, this paper do not select the IAE as the evaluation function. 

The ISE is the square of the error. Large errors are penalized more than smaller ones as the square of a large error is much bigger. Systems designated to minimize ISE tend to weaken large errors rapidly; however, they will have to tolerate small errors continuing for long periods. The gradual accumulation of small errors leads to low control accuracy in the later stage of ISE error calculation. 

The MSE improves the shortcomings of the ISE by calculating the mean of the ISE. However, the system needs to run for a long period of time in order to reduce the square of a large error calculated by the ISE. Therefore, the MSE is only applied in long-running systems. 

The ITAE, which is the absolute error multiplied by time, penalizes errors which exist after a long time, and is considered as a measure of system performance. The ITAE weights errors which exist after a long time and has an additional time multiplication in the error function, which emphasizes long duration errors and allows a faster response compared to the ISE and IAE. Thus, ITAE can solve problems more efficiently than other evaluation functions [43,44,45,46,47]. It has been shown that ITAE is a better evaluation function in the PID control system.

In this paper, double measurement value ITAE is selected as the evaluation function of a PID control system. The ITAE can be written as

(19) $$ITAE=\int_{0}^{\infty }t\left|e(t)\right|dt$$

Furthermore, the discrete ITAE is written as

(20) $$ITAE=\sum_{k=o}^{m}kT\left|e(k)\right|T$$

where *T* is the sampling period, *k* is the sampling number, and *m* is the total number of sampling times.

### 4.4. BAS-PID Control System 

**Figure 2:**
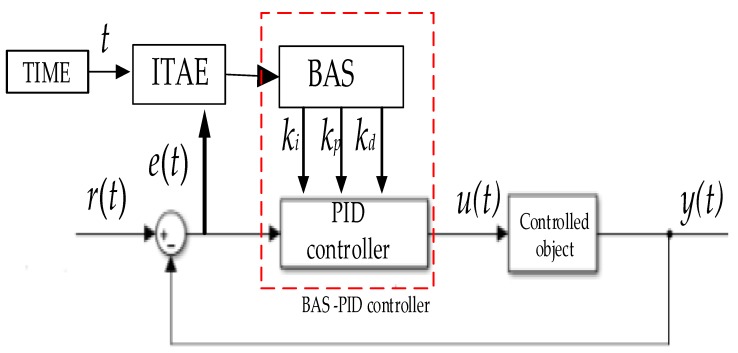
Block diagram of the BAS-PID controller.

The working principle of the BAS-PID controller is shown in Figure 2, where *u(t)* is the output signal of the BAS-PID controller, *y(t)* is the output signal from the control system, *r(t)* is the input signal to the control system, and *e(t)* is the error. PID parameters will be tuned automatically by BAS, after three PID parameter adjustment ranges are selected. BAS will tune three parameters according to the ITAE value of the control system in real time.

The control quality of the PID controller depends on three parameters. To obtain the optimal PID controller, in this paper, the PID parameter-tuning problem is converted into a class of three-dimensional parameter optimization questions. The three parameters are seen as the beetle’s position in three-dimensional space. The ITAE is regarded as the evaluation function. Beetle positions are randomly generated, BAS is run, and then the beetle positions are input into the PID controller as three parameters to calculate the evaluation function ITAE. The beetle position which minimizes the ITAE is considered to have the optimum PID parameters. The position of the beetle which minimizes the ITAE is used to update the optimum PID parameters in the current iteration. If the controllability of the PID control system meets the requirements of the engineering application or the searching procedure reaches the maximum number of iterations, the optimal position of the beetle will be chosen as the final PID parameters.

The parameter-tuning steps of the BAS-PID controller are as follows:

Step 1: Initialize all parameters and ranges. 

Randomly generate N beetle positions, $X_{n}^{T}$_(*n* = 1, 2, …, N)_ = [*K*_p_, *K*_i_, *K*_d_], where each parameter uses the real number coding. The discrete ITAE is regarded as the evaluation function. Set the maximum number of iterations, *T_MAX_*. Set c = −1 and T = 0. Set the initial sensing length, *d*^0^, and the initial step size, *δ*^0^.

Step 2: Normalize searching directions.

To expand the exploration environment, the searching directions of beetles can be normalized in random dimensional space using Equation (11). A random searching direction can be calculated as: $\vec{b}=\frac{rnd(3,1)}{\left\|rnd(3,1)\right\|}$_._

Step 3: Update the positions of the right and left antennae of one beetle.

Beetles do not know the precise location of food when foraging. They use their antennae to determine their next direction. When the antenna on one side is closer to food, the food odor received by the antenna is stronger, and the beetle will move to that side. The positions of the beetle’s right and left antennae are determined using Equations (12) and (13). The position of the right antenna can be obtained as $X_{nr}^{T}$ = $X_{n}^{T}$ + *d^T^*·$\vec{b}$, and the position of the left antenna can be obtained as $X_{nl}^{T}$ = $X_{n}^{T}$ *− d^T^*·$\vec{b}$.

Step 4: Update the next position of one beetle.

Operate the control system. The positions of the beetle’s right and left antennae, which can be seen as PID parameters, are carried over to the PID controller. Calculate the ITAE values of the evaluation function of the position of the right antenna, *f_ITAE_* ($X_{nr}^{T}$). Calculate evaluation function ITAE values of left antenna position: *f_ITAE_* ($X_{nl}^{T}$). Equation (14) is used to determine the next position of a beetle to obtain a new set of beetle positions.

Step 5: Calculate the ITAE of the evaluation function.

Operate the control system. Then, new beetle positions are input into the PID controller as three parameters to calculate the ITAE of evaluation function. By comparing all fitness values, the beetle position which minimizes the ITAE is used to determine the current optimum PID parameters in the current generation. Record the current optimum PID parameters and current minimum ITAE and use in the next step.

Step 6: Update the global optimum beetle position.

After comparing the current minimum ITAE with the previous minimum ITAE, the global optimum beetle position is updated, and the global optimum beetle position is chosen to be the optimal PID parameters.

Step 7: Update the sensing length of the antennae and the next step size.

The sensing length of the antennae can be updated using Equation (15): $d_{n}^{T}$ = 0.95·$d_{n}^{T-1}$ + 0.01. The step size of searching can be updated using Equation (16): $\delta_{n}^{T}$ = 0.95·$\delta_{n}^{T-1}$. By updating, the sensing length of the antennae and the step size of searching will be carried over to the next generation.

Step 8: Judge iterations. 

Calculate and judge whether iterations achieve terminating conditions. Calculate the number of iterations: *T* = *T +* 1. Judge whether iterations achieve the terminating condition *T* = *T_MAX_*. If *T* meets the terminating condition, the global optimum beetle position is chosen to be the optimal PID parameters. If *T* does not meet the terminating condition, return to Step 2 and start the next loop iteration.

Step 9: Stop.

Output the global optimum position of the beetle as the final three PID parameters. 

**Figure 3:**
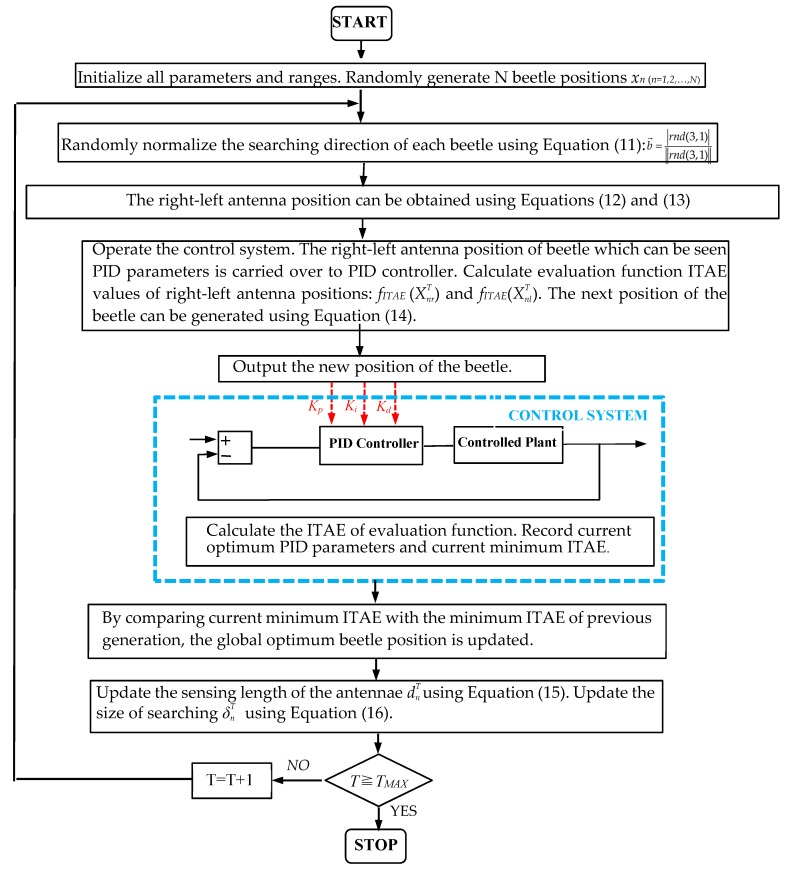
Parameters tuning flowchart of the BAS-PID controller.

The parameter-tuning flowchart of the BAS-PID controller is shown in Figure 3.

## 5. Simulation and Analysis

### 5.1. Simulation Environment

The electro-hydraulic servo valve is an FF102-30 (AVIC Nanjing Servo Control System Co., Ltd., Nanjing, China)., with a Ps of 21 MPa, a rated current of 50 mA, a *ξ_sv_* of 0.5, a *K_sv_* of 0.006 m^3^/(sA), and a no-load flow of 2.315 × 10^−4^ (m^3^/s). The saturation value of the servo amplifier control voltage is ±10V, the length of the piston displacement is 35 mm, the piston rod area is 0.001 m^2^, the position sensor gain is 50 V/m, the position sensor range is 7100 mm, the stroke of the cylinder is 200 mm, the rated flow of the cylinder is 30 L/min, and the oil supply pressure is 4.5 MPa.

All the model parameters of the electro-hydraulic position servo control system were obtained by identifying system parameters. The identification techniques include the product look, numerical derivation, experimental testing, and engineering experience [48,49,50,51]. The natural parameters of the electro-hydraulic servo valve in the model are obtained from the servo valve product book. The natural frequency, damping, and conversion factor of the servo valve were obtained from dynamic characteristic test curves by numerical derivations. Natural parameters of the servo amplifier and the position sensor were obtained from the product book. Moreover, structural parameters, including the effective piston area of the servo cylinder, the total piston stroke of the servo cylinder, and the volume of the oil pipe were obtained from the factory data of the cylinder. The working parameters—including system supply oil pressure, system return oil pressure, and sensor gain—were obtained from the experimental testing. Other parameters were selected based on engineering experience.

The viscous damping coefficient can be ignored. Therefore, the model of the controlled object obtained by identification is as

$$G(s)=\frac{4.63}{4.528\times 10^{-12}s^{5}+4.1988\times 10^{-9}s^{4}+5.1725\times 10^{-6}s^{3}+0.002s^{2}+s}$$

To verify the performance of BAS, this paper also compared the result of the proposed BAS-PID controller with those of PID controllers based other popular artificial intelligence algorithms, including PSO, GA, and FA.

For PSO, parameters were set as: learning factors *c*_1_ = *c*_2_ = 1, inertial weight *w* = 1.

For GA, roulette wheel selection with an elitism mechanism was used, and parameters were set as: crossover probability P_cross_ = 0.7, mutation probability P_m_ = 0.02. 

For FA, parameters were set as: initial attractiveness *β_0_* = 1.

For BAS, parameters were fixed as: initial sensing length of the antennae *d^0^* = 2, the initial step size δ^0^ = 1.

For all algorithms, the population size was set as 50 and the maximum number of evaluations was set as 200.

All of the initial population positions of the different algorithms were generated from a uniform distribution. The lower and upper bounds of the search space of three parameters were given by (0, 100). Each optimization method was implemented over 10 independent runs in the MATLAB software (MathWorks, Natick, MA, USA).

The input and disturbance signals were step signals. Through the iteration of the ITAE, the process of PID parameter-tuning can be seen as finding the solution which minimizes the ITAE value. The total sampling time was 100 and the sampling period was 0.01 s.

### 5.2. Simulation Results and Analysis

Table 3 shows the results of the ITAE, overshoots M_p_, the rise time *t_r_*, the settling time *t_d_* and their corresponding PID controller parameters. The overshoot M_p_ reflects the stability of the system, the rise time *t_r_* reflects the response capability of the system, and the settling time *t_d_* reflects the adaptability of the system. As seen from Table 3, the BAS parameter-tuning method has the lowest overshoot, the fastest rise time and settling time, and the smallest value of ITAE of all algorithms.

**Figure 4:**
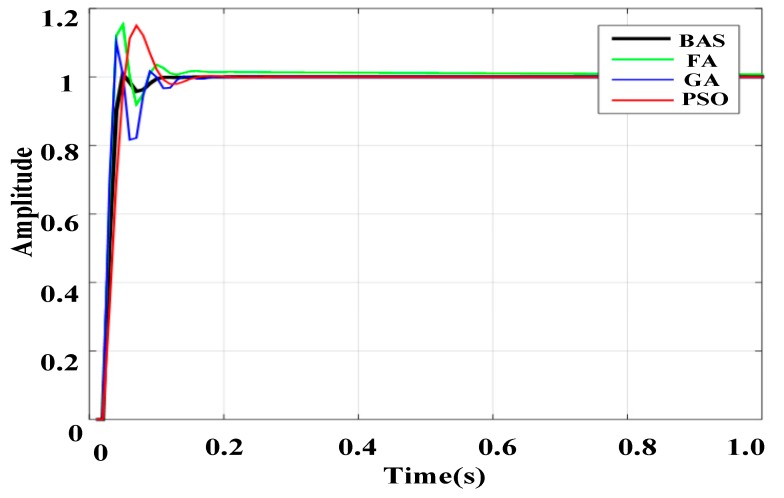
Response curves of the step signal.

Response curves derived from the step input are shown in Figure 4. From the figure, it can be seen that, compared with PIDs using other algorithms, BAS-PID shows the best performance. The step response curve of the BAS -PID controller converges to the desired set value with the minimum overshoot and least steady time. Using the BAS-PID controller, the system can maintain high dynamic characteristics and stability precision, and the performance of the system is unaffected by outside interference signals, showing perfect robustness.

**Figure 5:**
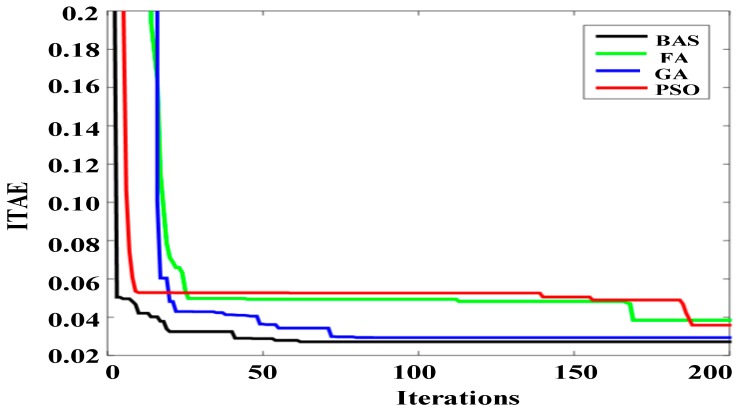
Average convergence curves of ITAE.

Figure 5 illustrate the average convergence curves of all algorithms disposing ITAE functions over 10 independent runs. As can be observed in the Figure 5, BAS has the fastest convergence speed in all algorithms, showing better performance when optimizing PID controller parameters. Additionally, BAS achieved the highest iteration speed of the four algorithms. The BAS convergence curve tends to accelerate as the number of iterations increases, and quickly converges towards the optimal value after completing almost half of the iterations. BAS demonstrates outstanding convergence stability and competence in jumping out from limited optimum.

**Figure 6:**
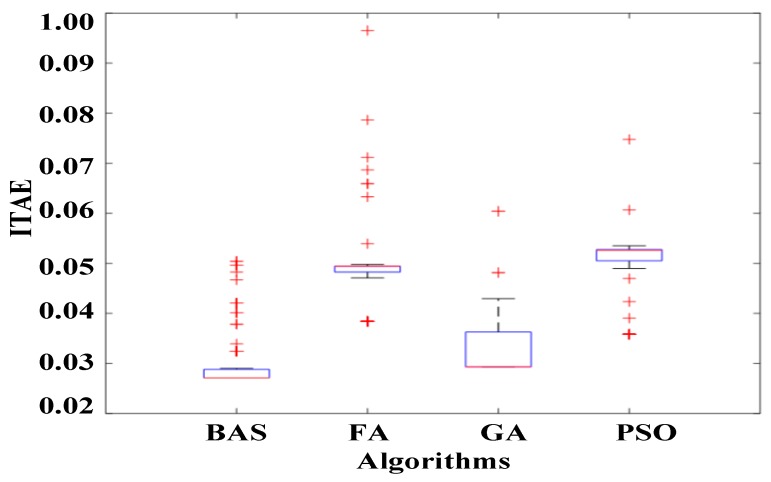
Boxplot of ITAE.

The box plot is shown in Figure 6 displays a set of scattered data. The stability of the control system can be shown by examining the position of the median, upper quartile, and lower quartile in the boxplot. The boxplot measures the dispersion of the ITAE value by graphical means. We note that the value of ITAE calculated by BAS has fewer outliers and a lower degree of dispersion than other algorithms, indicating that the proposed BAS has excellent stability. The median, upper quartile, and lower quartile of the ITAE calculated by BAS are lower than for other algorithms, which proves that BAS has excellent optimization capability.

**Figure 7:**
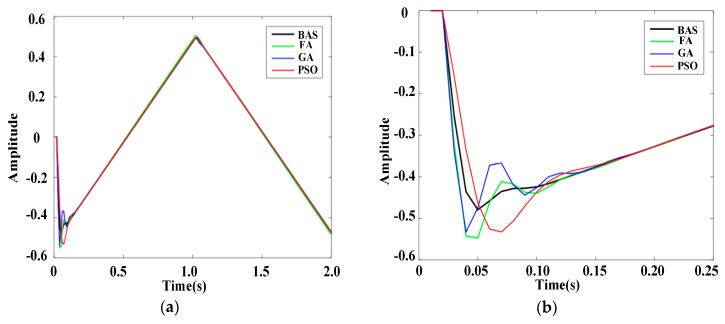
Response curves of the triangular wave signal. (**a**) Whole response curves. (**b**) Local amplification curves.

**Figure 8:**
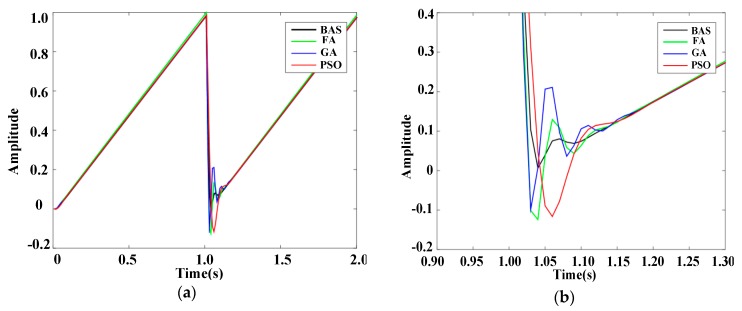
Response curves of the sawtooth wave signal. (**a**) Whole response curves. (**b**) Local amplification curves.

To further prove the reliability of the BAS-PID controller, response results of PID controllers based on different algorithms are presented in Figure 7 and Figure 8, when disturbance signals are the triangular wave signal and the sawtooth signal, respectively. Figure 7a shows the response curves of the triangular wave signal, and Figure 7b shows the local amplification of the response curves. Under the control of the BAS-PID controller, the amplitude error is the smallest and the smoothing speed is the highest. Figure 8a shows the response curve of the sawtooth wave signal, and Figure 8b shows the local amplification of the response curve. Figure 7 shows that the BAS-PID controller can not only restrain the disturbance signal but also enhance the dynamic characteristics and the steadiness of the robustness. For the two different interference signals, the system has a remarkable capability to maintain the stability and reduce the shaking and concussion when the BAS-PID controller is selected, and the performance of the system is unaffected by external interference.

## 6. Experimental Analysis

**Figure 9:**
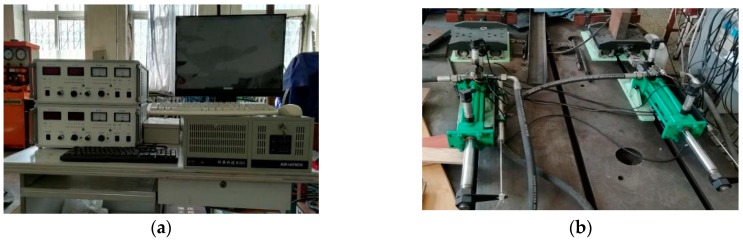
The electro-hydraulic semi-physical experiment platform. (**a**) Experiment platform. (**b**) Electro-hydraulic servo system.

To certify the effectiveness of the proposed BAS-PID controller in an actual working environment, the designed controller was used in a comprehensive experiment of the operation of the electro-hydraulic position servo control system. The electro-hydraulic semi-physical experimental platform is illustrated in Figure 9.

Under the experimental conditions, the power of the hydraulic source was 5.5 k, the rated flow rate was 30 L/min, the rated pressure was 5 MPa, an ADVAN-TECH PCL1710HG multi-function board was selected, the sampling time was 0.01 s, and the rated current was 40 mA.

**Figure 10:**
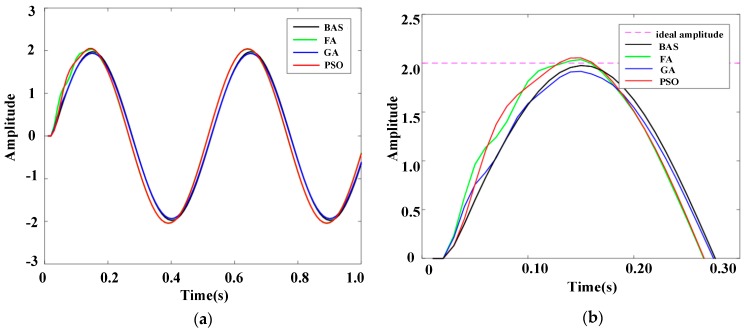
Response curves of the sinusoidal signal whose amplitude is 2. (**a**) Whole response curves. (**b**) Local amplification curves.

**Figure 11:**
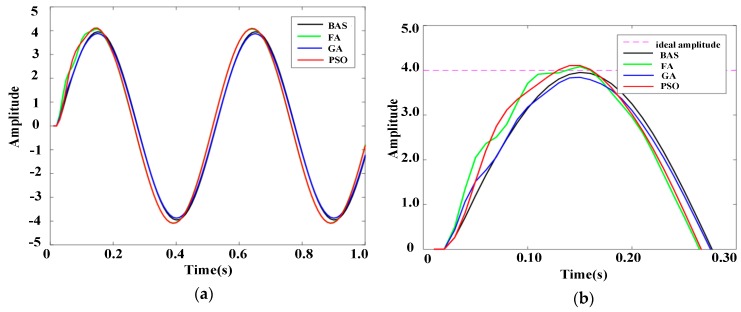
Response curves of the sinusoidal signal whose amplitude is 4. (**a**) Whole response curves. (**b**) Local amplification curves.

**Figure 12:**
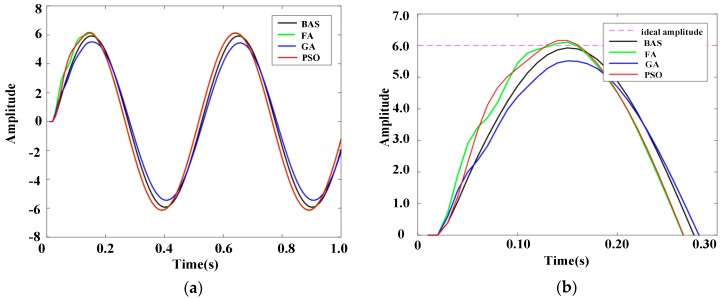
Response curves of the sinusoidal signal whose amplitude is 6. (**a**) Whole response curves. (**b**) Local amplification curves.

**Figure 13:**
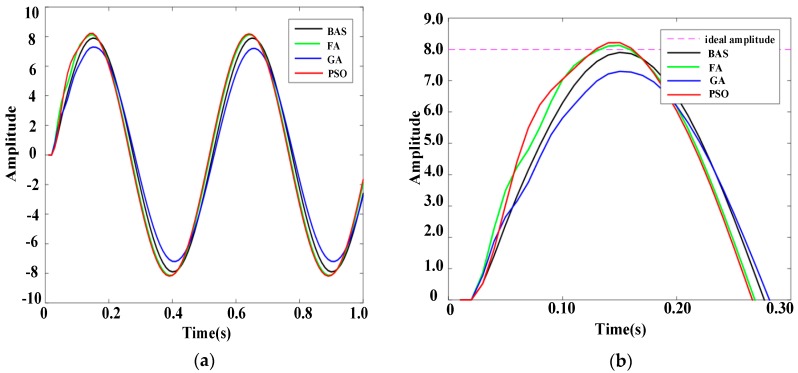
Response curves of the sinusoidal signal whose amplitude is 8. (**a**) Whole response curves. (**b**) Local amplification curves.

**Figure 14:**
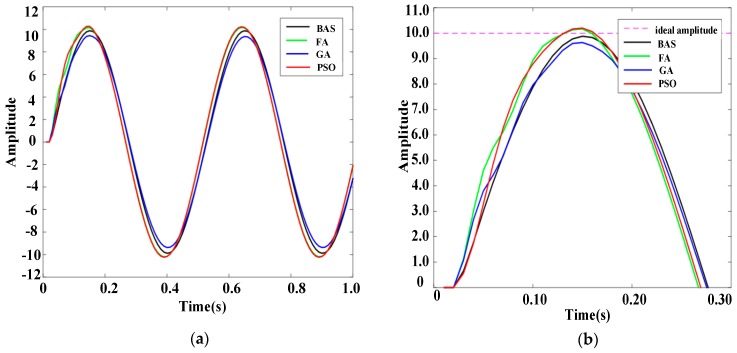
Response curves of the sinusoidal signal whose amplitude is 10. (**a**) Whole response curves. (**b**) Local amplification curves.

A sinusoidal signal is usually chosen as the performance testing signal. The frequency response characteristic of the system under a sinusoidal signal can be implemented to test the system performance. The frequency response, i.e., the response of the system under a sinusoidal signal, can be used to determine the resonance frequency, impedance, dynamic stiffness, and vibration stability of the system. The amplitude frequency characteristic, *A_ω_*, which is defined as the amplitude ratio of the input signal to the ideal signal, is invoked as the index in the frequency response. If *A_ω_* is closer to 1, the system is more stable. In the experiment, five sinusoidal signals with different amplitudes were used as disturbance signals. For the five sinusoidal signals, the angular velocity was 4π, the initial phase was zero, and the amplitudes were 2, 4, 6, 8, and 10, respectively. The PID controllers based on different algorithms, including FA, GA, and PSO, were selected for experiments in the same experimental environment. The ranges of PID parameters for all algorithms and iterations were the same as in Section 5. The experimental results were contrasted and analyzed. The response results are shown in Figure 10, Figure 11, Figure 12, Figure 13 and Figure 14. 

Figure 10b, Figure 11b, Figure 12b, Figure 13b, and Figure 14b show the local amplification curves for the five sinusoidal signals. It can be seen from Figure 10, Figure 11, Figure 12, Figure 13 and Figure 14 that the response curves for the BAS-PID controller have the smallest distance between the ideal amplitude and the actual amplitude. Therefore, we can infer that the BAS-PID controller has outstanding performance including vibrational stability, strong dynamic stiffness, and high mechanical impedance.

Frequency response indices for different PID controllers are listed in Table 4, where *∆Aω* = |1 – *Aω*|. From Table 4, it can be seen that the frequency response of the system controlled by the BAS-PID controller still is clearly superior to the other PID controllers. The amplitude frequency characteristic Aω of the system controlled by the BAS-PID controller is the closest to 1. This shows that the BAS-PID controller can asymptotically maintain system stability when the interference signal is altered dramatically.

**Figure 15:**
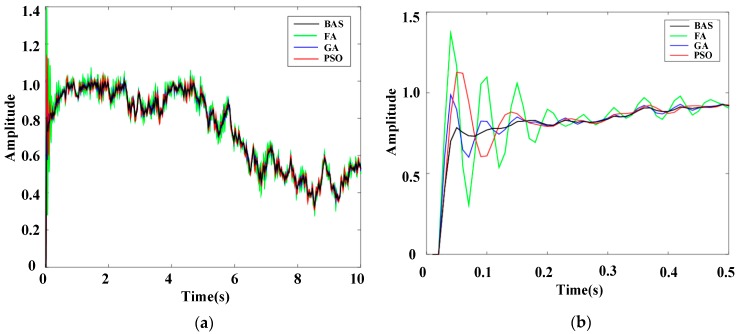
Response curves of the random signal l. (**a**) Whole response curves. (**b**) Local amplification curves.

**Figure 16:**
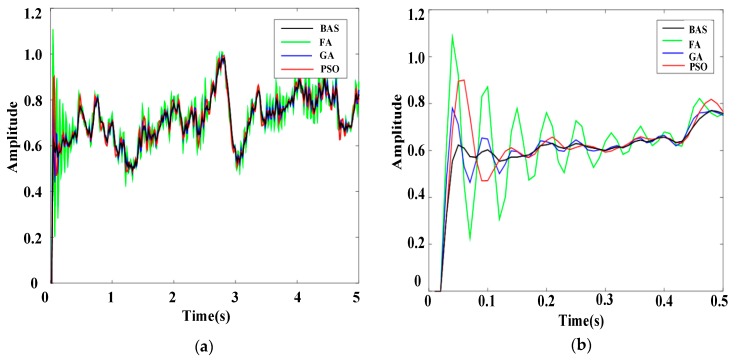
Response curves of the random signal 2. (**a**) Whole response curves. (**b**) Local amplification curves.

To further display the performance of the BAS-PID controller, the response results of the system under different PID controllers when interference signals were random signals are presented in Figure 15 and Figure 16.

Local enlarged drawings clearly show that the BAS-PID controller can not only rapidly suppress interference signals but also prevent excessive overshoot. For the unusual interference signals, the BAS-PID controller has a remarkable capability to maintain the system stability and reduce the shaking, and the performance of the system is unaffected by external interference. In other words, the BAS-PID controller has anti-interference ability and provides remarkable balance, which can enhance the anti-seismic properties of the electro-hydraulic position servo control system in an unknown environment.

## 7. Conclusions

In order to enhance the control accuracy and ability of an electro-hydraulic position servo control system, the paper addressed the problem of determining three parameters of PID controllers. A PID parameter tuning method based on the beetle antennae search algorithm was applied to an electro-hydraulic position servo control system. A transfer function model was obtained by system parameter identification. A basic mathematical model of the electro-hydraulic position servo control system was established through theoretical analysis. The PID tuning problem was converted into a three-dimensional optimization question. The performance of the BAS tuning method was tested by ITAE and compared with that of the PSO, GA, and FA algorithms. An analysis of the performance of the BAS-PID controller with the electro-hydraulic position servo control system showed that the BAS algorithm can effectively adjust three parameters of the PID controller. The BAS-PID controller can bring many advantages, such as restraining system interference and meeting the requirement that the control system can maintain the robustness when there are the different external signals, which can better maintain the control needs of the electro-hydraulic position servo control system.

## Figures and Tables

**Table 1:** Variant ways and advantages of BAS variants.

BAS Variants	Variant Way	Advantages
BPNN-BAS [30]	• BAS was used to train back propagation neural network (BPNN)	• High BPNN training speed • Make weights optimal
PSO-BAS [32,38,39]	• BAS was combined with PSO	• Greater global search ability • Better searching capacity than that of standard PSO
BAS-MkRVR [33]	• BAS was used to select the appropriate kernel parameters and controlled parameters of the mixed kernel relevance vector regression (MkRVR)	• Stronger prediction capacity than RBFRVR and Sigmoid RVR.
SVM-BAS [34]	• The hyper-parameters of the support vector machine (SVM) were firstly tuned by BAS	• Less time-consuming • Low-cost • Non-destructive
ESVR-BAS [35]	• The hyper-parameters of the evolved support vector regression was tuned by BAS	• More efficient than random hyper-parameter selection • High predictive capability
BAS-WPT [36]	• Normalization method and penalty function method were used to extend BAS	• Not require prior parameter tuning • Simple to design, implement, and has less complexity
OABAS [37]	• OABAS was designed on the basic of BAS to improve the path planning	• Wider search range and the breakneck search speed • Shorter path length

**Table 2:** Uniqueness of BAS among algorithms.

Contrastive Algorithms	Advantages of BAS	Relevant Recent References
PSO	Faster iteration speed Stronger ability to jump local optimal solution	[32,37,38,39]
GA	Does not need binary to represent decimal numbers The BAS program runs faster	[39]
FA	Does not need more initial parameters BAS program code simple	This paper
BA	Simple to implement, and has less complexity	[42]
ABC	Higher efficiency Lower time complexity	[37]

**Table 3:** PID parameters and performances of the control system.

	BAS	FA	GA	PSO
K_p_	7.9927	9.5773	8.0156	8.2923
K_i_	0.1412	6.9449	0	0.0225
K_d_	0.0532	0.0714	0.0978	0
ITAE	0.0275	0.0384	0.0294	0.0358
M_p_	0.0067	0.1439	0.1015	0.1503
t_r_	0.022	0.051	0.056	0.029
t_d_	0.023	0.026	0.029	0.036

**Table 4:** Frequency response characteristics.

Amplitude	Index	BAS	PSO	GA	FA
2	*A_ω_*	0.9870	1.0270	0.9695	1.0165
	*∆A_ω_*	0.0130	0.0270	0.0305	0.0165
4	*A_ω_*	0.9878	1.0267	0.9693	1.0175
	*∆A_ω_*	0.0122	0.0267	0.0307	0.0175
6	*A_ω_*	0.9878	1.0271	0.9186	1.0177
	*∆A_ω_*	0.0122	0.0271	0.0814	0.0177
8	*A_ω_*	0.9879	1.0267	0.9878	1.0174
	*∆A_ω_*	0.0121	0.0267	0.0122	0.0174
10	*A_ω_*	0.9878	1.0191	0.9696	1.0190
	*∆A_ω_*	0.0122	0.0191	0.0304	0.0190
